# Influence of university agricultural technology extension on efficient and sustainable agriculture

**DOI:** 10.1038/s41598-024-55641-1

**Published:** 2024-02-28

**Authors:** Zhaoli Dai, Qing Wang, Jiyu Jiang, Yan Lu

**Affiliations:** 1 School of Economics and Management, Anhui Agricultural University, Hefei, China; 2 The Centre for Research on Science Technology and Education of Agriculture, Anhui Agricultural University, Hefei, China

**Keywords:** Evolution, Plant sciences

## Abstract

Agricultural extension, as an important part of modern agriculture, can promote the scientific transformation of the traditional agricultural production model. This paper analysed the impact of university agricultural technology extension on efficient and sustainable agriculture using difference-in-differences model (DID). The results showed that university agricultural technology extension plays a facilitating role by influencing the coordinated development and green development dimensions in efficient and sustainable agriculture; there is a moderating effect of modern agricultural industrial parked in university agricultural technology extension and efficient and sustainable agriculture; there are significant differences in the impact of university agricultural technology extension on efficient and sustainable agriculture across regions and different levels of development. The findings have important implications for evaluating the effectiveness of current university agricultural extension policies and how to further promote university agricultural extension. The study also established an evaluation index system for efficient and sustainable agriculture, explored the mechanism of university agricultural extension in promoting efficient and sustainable agriculture, and enriched relevant theoretical research.

## Introduction

Technological innovation and entrepreneurship play an important role in driving economic and social development^1^. Agricultural technology extension is crucial to alleviating the resource and environmental constraints faced by developing countries and transforming the mode of agricultural development. With the advancement of agricultural technology extension, extension entities are becoming more diverse. Universities are utilizing their technological and R&D advantages to conduct practical trials to further agricultural technology extension. The objective is to encourage efficient and sustainable development of local agriculture using this technology extension approach. As the world's largest developing country, China has entered a new normal of economic development, and its macroeconomic goal has shifted from high-speed growth to high-quality growth. As the main pillar industry, the development of efficient and sustainable agriculture has become the key task of China in the new period, but the international situation it faces is extremely severe. With the deepening of globalization, on the one hand, developed countries are squeezing and controlling the development of China's agricultural industry by taking high-level agricultural technology and agricultural subsidies as carriers and relying on capital flow and market expansion^2^. On the other hand, China faces resource and environmental constraints and inefficient resource management and use^3^. Therefore, China urgently needs to change its development mode through agricultural technology, push agriculture to the path of efficient and sustainable development, and finally realize the transformation from a large agricultural country to a powerful agricultural country. The investigation of Chinese universities' role in agricultural technology extension is highly significant in facilitating the effective application of agricultural science and technology, transforming conventional agricultural practices and ultimately supporting the efficient and sustainable development of regional agriculture. Anhui Agricultural University is among the first 10 pilot projects in China to adopt the new model of university agricultural technology extension. Anhui Agricultural University is an exemplar of the "one-stop, one-alliance, one-centre" typology for agricultural technology extension, this research focuses on the following questions: Does the extension of agricultural technology in universities help push agriculture towards efficient and sustainable development? If so, what is its specific mechanism of action?

Some scholars have found that socialized service plays an increasingly important role in improving agricultural productivity and increasing farmers’ income, which can promote efficient and sustainable development^4^. Anhui Agricultural University’s “one station, one alliance, and one center” new university agricultural technology extension is only a pilot policy. Since 2012, Anhui Agricultural University has established eight comprehensive experimental stations in Dabie Mountain, central Anhui, eastern Anhui, southwestern Anhui, northwestern Anhui, southern Anhui, northern Anhui, and the Yangtze-Huai river basin. The differences of this policy pilot in different regions and years constitute a “quasi-natural experiment.” Therefore, this research aims to explore the influence and mechanism of university agricultural technology extension on efficient and sustainable agriculture.

## Literature review

The references closely related to the research question can be divided into two main branches. Existing research on agricultural extension in universities focuses primarily on two topics: agricultural extension and efficient, sustainable agriculture. The first branch is the research on agricultural technology extension. Agricultural technology extension has been studied from three aspects. Firstly, the impact analysis of agricultural technology extension on agricultural production. Some scholars have found that agricultural technology extension plays a positive role in improving the productivity of agricultural production and technology adoption by farmers^5,6^. Second, to study the construction of agricultural technology extension systems. To build and improve agricultural technology extension systems, it is first necessary to develop a critical mass of new generation, committed and well-trained men and women to serve the agricultural sector^7^. Third, the mode of agricultural technology extension should be explored. In the process of exploring the mode of agricultural technology extension, many innovative achievements have been made. Therefore, some scholars have analyzed the influence of the type and mode of agricultural technology extension on technical efficiency in the transition period^8^.

The second branch concentrates on efficient and sustainable agriculture. In the existing literature, research on sustainable agriculture is mainly concerned with the facilitating effects of modern agricultural technology on sustainable agriculture. Sharma et al. showed how knowledge-based agriculture can improve sustainable productivity^9^. Singh et al. critically highlighted the material application of NPs and pointed out the crucial gaps in the use of nanotechnology for sustainable agriculture^10^. Pereira et al. explore the potential applications of lignin nanoparticles in the agricultural sector^11^, highlighting the lignin extraction processes, nanoparticle production methods, biological activity analysis and emerging applications relevant to sustainable agriculture. In terms of constructing a measurement indicator system for sustainable agriculture, Zhang et al. consider that agricultural production cannot be achieved without natural resources such as arable land, water and the environment^3^.

While the literature has examined the impact of agricultural technology extension on agricultural production and the creation of sustainable agriculture indicators, scholars have generally agreed that the extension of technology is crucial to the high-quality advancement and maintainable growth of the industry. Additionally, it is believed that the extension of technology enhances accounting and auditing practices in the agricultural sector, resulting in a beneficial influence on such activities in the sector^12^. The utilization of mobile phone technology amplifies farmers' capacity for agribusiness and has the potential to enhance agribusiness performance^13^. Nevertheless, the literature on the impact of agricultural extension programs in universities towards sustainable and effective agricultural development, their mechanism of action, and the contribution of modern agricultural industrial parks, lacks comprehensive and in-depth analysis. This paper concentrates on the impact mechanism of promoting agricultural technology by universities in achieving efficient and sustainable agricultural development, the moderating effects of contemporary agricultural industrial parks, and the disparities in the role of university agricultural technology promotion in various regions. The study fills some gaps in existing research by distinguished scholars. Compared with the existing literature, the possible contributions of this research are as follows. First, the entropy method is used to calculate the efficient and sustainable agriculture of 90 counties and districts in Anhui Province, China during 2008–2020 from three dimensions: coordinated development, efficient development and green development. Then, the influence of university agricultural technology extension on efficient and sustainable agriculture is explored, which provides an empirical basis for the necessity of university agricultural technology extension. Second, the action mechanism of university agricultural technology extension on efficient and sustainable agriculture is discussed from the specific influences of university agricultural technology extension in three dimensions, namely coordinated development, efficient development and green development, and the modulating effect of modern agricultural industrial parks. Third, the heterogeneity of the influence of university agricultural technology extension on efficient and sustainable agriculture is explored from two aspects: three regions (northern, central, and southern Anhui) and the local agricultural development foundation. This research provides an empirical reference from China for perfecting the university agricultural technology extension system and developing efficient and sustainable agriculture.

## Methods

### Difference-in-differences model (DID)

Difference-in-differences model (DID) is a quasi-natural experimental method currently used in policy evaluation. It is based on the same principle of dividing the sample into a treatment group that is affected by the policy and a control group that is not affected by the policy, and then comparing the treatment group with the control group using counterfactual thinking to infer the effect of the policy. The prerequisite for employing the DID model stipulates that the introduction of the policy under scrutiny has an impact on certain samples but not others, with the former forming the treatment group and the latter constituting the control group. Anhui Agricultural University functions as a university agricultural technology extension through the establishment of extensive agricultural experimental stations. Consequently, the policy of university agricultural technology extension will impact areas with comprehensive agricultural experimental stations, while those without such amenities shall remain unaffected. In this study, the sample was selected from all municipalities and counties in Anhui Province, with the treatment group being the areas with an integrated agricultural experiment station built by Anhui Agricultural University, and the control group being other areas without an integrated agricultural experiment station.

The use of the DID method for policy evaluation can remove the interference of other factors, such as the macroeconomic environment, other than policy shocks. In this study, since the development of efficient and sustainable agriculture in a region may be influenced by other potential factors such as the level of regional economic development and the level of resources, the use of this method can accurately identify the policy impact of university agricultural technology extension on efficient and sustainable agriculture and obtain more scientific results. The prior application of the parallel trend test guaranteed that both the treatment and control groups exhibited equal progress in effective and sustainable agricultural development before encountering any shocks resulting from the agricultural extension policy of the university. As a result, all the prerequisites for utilizing the DID method were met. Furthermore, to alleviate the impact of sample selection bias and other unobserved random factors, this paper utilises propensity score-matching double-difference (PSM-DID) and placebo tests to guarantee the dependability of the test outcomes.

### Analytical framework

Under the policy advocacy and active practice of universities, agricultural technology extension in universities can play an important role in the development of efficient and sustainable agriculture. On the one hand, colleges and universities are at the nexus of the first productivity of science and technology, the first resource of talents, and the first force of innovation. Their research results can provide new methods and technologies for local agricultural development and provide technicians and technical training for local agricultural production through the cultivation and transportation of agricultural professionals. Therefore, with the rich resources of colleges and universities and the local development conditions, local agriculture can be put on the track of efficient and sustainable development. On the other hand, the development of efficient and sustainable agriculture depends on government support, including policy guidance, capital investment and infrastructure construction. Agricultural technology extension in colleges and universities is supported by local governments in many aspects, such as policies and funds, through the cooperation and co-construction mode between colleges and universities and local governments, which helps to build the platform of agricultural technology extension in colleges and universities. Efficient and sustainable agriculture is promoted through experiments, demonstrations, training, information and technical support by relying on the platform of agricultural technology extension in universities and a complete extension system.

From the specific action mechanism, efficient and sustainable agriculture can be promoted through agricultural technology extension in universities through the following aspects. First, it improves the level of coordinated agricultural development. Agricultural technology extension in universities provides guidance and technology for the adjustment of local agricultural industry structure, promotes the adjustment of industrial structure, develops local characteristic breeding industry and builds local characteristic agricultural product brands, and facilitates the coordinated development of agricultural industry, thereby promoting efficient and sustainable agriculture^14^. Second, it improves the level of efficient agricultural development. Agricultural technology extension in universities can improve the production level or quality of crops using local advanced agricultural technology, which can reduce the losses caused by improper practices in the production process, realize the efficient development of agriculture, and promote efficient and sustainable agriculture. Thirdly, the green development level of agriculture will be improved. The key technologies of agricultural green production, such as fertilizer reduction and Sulphur-free fermentation^15^, which are involved in the extension of agricultural technology in universities, can reduce environmental damage and resource loss in the process of local agricultural production, help improve the green development level of agriculture, and promote efficient and sustainable agriculture. In addition, modern agricultural industrial parks are a modern agricultural development platform based on large-scale planting and breeding bases and driven by industrialized leading enterprises^16^, which can play a strong role in promoting efficient and sustainable agriculture. Agricultural technology extension in universities can expand its influence by supporting the establishment of modern agricultural industrial parks. It can also rely on industrial parks to provide more services, such as agricultural technology extension and farmer training, and exert the agglomeration effect of industrial parks. Finally, it can enhance the facilitating effect of agricultural technology extension in universities on efficient and sustainable agriculture.

University agricultural technology extension may influence efficient and sustainable agriculture differently due to differences in resource endowment and regional agricultural development foundation. On the one hand, the difference in geographical location leads to an uneven distribution of resources, which are partially concentrated in the central and southern parts of Anhui Province, creating a better external environment for efficient and sustainable agriculture in this region. However, the northern part is a traditional agricultural development area with a large proportion of rural population and a large economic gap between urban and rural areas. This area also has a weak economic base, backward agricultural infrastructure and a poor external environment compared to the central and southern parts, which may hinder the expansion of agricultural technology extension in universities. On the other hand, the regions with a weak development base are less willing to adopt new technologies and have a weak ability to apply new technologies. The extension of agricultural technology in universities has no significant effect on the development of high-quality agriculture in this region, and the regions with a good development foundation are more likely to be positively influenced by the extension of agricultural technology in universities.

### Data

County-level data for Anhui Province, China, from 2008 to 2020 are taken from the *Anhui Statistical Yearbook* and the *China County Statistical Yearbook* throughout these years. The supplementary data of each district and county from 2008 to 2020 are obtained from the statistical yearbooks of various prefecture-level cities. The missing data of some years in the above statistical yearbooks are supplemented by interpolation. After removing the areas with severe data loss and administrative changes, the data in this paper cover 90 areas and 878 observations.

### Empirical model

#### Influence of university agricultural technology extension on efficient and sustainable agriculture

In 2012, Anhui Agricultural University launched the pilot project of university agricultural technology extension in Anhui Province. It selected Jinzhai District of Lu'an City, Lujiang District of Hefei City, Mingguang City of Chuzhou City, Huaining District of Anqing City, Linquan District of Fuyang City, Huangshan District of Huangshan City, Yongqiao District of Suzhou City and Dingyuan District of Chuzhou City as the pilot areas. The university established comprehensive agricultural experiment stations in the above areas to provide technical guidance and talent incubation services for local agricultural development. From the perspective of policy evaluation, the extension of agricultural technology in universities can be regarded as a quasi-natural experiment. The pilot areas serve as the treatment group and the other areas in Anhui Province as the control group. In this research, the difference-in-differences (DID) method is used to analyze the influence of university agricultural technology extension on efficient and sustainable agriculture. The econometric model is as follows:

1 $$ ESA_{{{\text{it}}}} = \alpha_{0} + \alpha_{1} policy_{it} + \gamma Z_{it} + \delta_{i} + \varphi_{t} + \varepsilon_{it} $$

where $ESA_{it}$ is the comprehensive index of efficient and sustainable agriculture, which is calculated by entropy method; $policy_{it}$ is a virtual variable for agricultural technology extension in universities, which is taken as 1 for the pilot area in the year when or after the agricultural comprehensive experimental station is established, and 0 otherwise; $Z_{it}$ is a control variable that affects efficient and sustainable agriculture; $\delta_{i}$ is the regional fixed effect; $\varphi_{t}$ is the fixed effect of the year; $\varepsilon_{{i{\text{t}}}}$ is a random disturbance term; and subscripts *i* and *t* denote the region and year, respectively. Therefore, $\alpha_{1}$ in Formula (1) measures the influence of university agricultural technology extension on efficient and sustainable agriculture. If its coefficient is significantly positive, then the university agricultural technology extension is helpful in promoting efficient and sustainable agriculture.

#### Parallel trend test and dynamic influence of university agricultural technology extension on efficient and sustainable agriculture

An important premise of the DID method used in policy evaluation is that the outcome variables used in the treatment and control groups meet the parallel trend before being influenced by the policy. In addition, the policy effect of university agricultural technology extension may not be obvious in a short time, so the dynamic influence of university agricultural technology extension on agricultural efficiency and sustainability needs to be analyzed. To test the parallel trend hypothesis and analyze the dynamic influence of university agricultural technology extension on efficient and sustainable agriculture, the following econometric model is constructed:

2 $$ ESA_{it} = \alpha_{0} + \alpha_{1} policy_{it}^{ - 5} + \alpha_{2} policy_{it}^{ - 4} + \cdots + \alpha_{12} policy_{it}^{6} + \gamma Z_{it} + \delta_{i} + \varphi_{t} + \varepsilon_{it} $$

where $policy_{it}^{ \pm j}$ is a series of dummy variables, which is taken as 1 for pilot areas in the first *j* years before the year when the agricultural comprehensive experimental station is established, and 0 otherwise; and the year when the comprehensive agricultural experimental station built is the reference group. The meanings of other variables are the same as those in Formula (1). Given the short period before the pilot extension of agricultural technology in the university in the sample, five years before and six years after the implementation are selected. In Formula (2), $policy_{it}^{ - j}$ measures whether a significant difference exists in the change trend of agricultural high-quality development level between the pilot area and other areas in Anhui Province before the university agricultural technology extension. If the coefficient is insignificant, then the change in the efficient and sustainable agriculture level between the pilot area and other areas in Anhui Province before the university agricultural technology extension meets the parallel trend. $policy_{it}^{ + j}$ measures the dynamic influence of university agricultural technology extension on efficient and sustainable agriculture.

### Variables

In this research, the explained variable is the comprehensive index of efficient and sustainable agriculture (ESA), which is solved by entropy method. In this research, a set of evaluation index system of county-level efficient and sustainable agriculture is constructed, which includes 3 dimensions and 10 first-level indexes, by referring to previous research results^17,18^ and considering the availability and continuity of data, see Table 1.

**Table 1:** Evaluation index system of efficient and sustainable agriculture.

Criterion layer	Indicator layer	Variable definitions	Attribute	Units
Coordinated development	Planting structure	1 − (grain planting area/ total crop planting area)	+	−
	Agricultural economy coordination level	Agricultural added value/(agricultural, forestry, and animal husbandry and fishery added value − agricultural added value)	+	−
Efficient development	Labor productivity of primary industry	Value added in the primary industry/employees in the primary industry	+	10^4^ yuan /person
	Grain yield per unit area	Total grain yield/sown area of grain crops	+	t/hm^2^
	Output of livestock products per labor	Total meat output/employees in the primary industry	+	t/person
	Agricultural land output rate	Total agricultural output value/sown area of crops	+	10^4^ yuan/hm^2^
Green development	Fertilizer application amount per unit area	Fertilizer application rate/sown area of crops	-	t/hm^2^
	Pesticide usage per unit area	Pesticide usage/sown area of crops	−	t/hm^2^
	Agricultural film usage per unit area	Agricultural film usage/sown area of crops	−	t/hm^2^
	Agricultural CO_2_ emissions	Total CO_2_*(agricultural, forestry, and animal husbandry and fishery output value/GDP)	−	million tons

The main calculation steps are as follows. First, given that the dimensions and orders of magnitude of each evaluation index in the evaluation system are not completely consistent, the original data are standardized to eliminate the influence of dimensional differences and orders of magnitude differences. Second, the entropy method in the objective weighting method is adopted to determine the weight of each evaluation index in the evaluation system of efficient and sustainable agriculture. Third, the multi-objective linear weighting function method is used to weigh all the evaluation indexes, and the comprehensive index of efficient and sustainable agriculture and the index of each criterion layer are obtained.

### Data standardization–extremum method

The extremum method is used to standardize the positive and negative indexes in the evaluation system, as shown as follows:

3 $$ {\text{Positive indexes}}{:}\;\;Y_{ij} = \frac{{x_{ij} - x_{i,\min } }}{{x_{i,\max } - x_{i,\min } }}{\text{; Negative indexes:}} \;\; Y_{ij} = \frac{{x_{i,\max } - x_{ij} }}{{x_{i,\max } - x_{i,\min } }} $$

where $Y_{ij}$ is the standardized index value; $x_{ij}$ represents the original data of index *j* of district/county *i*; and $x_{i,\max }$ and $x_{i,\min }$ are the maximum and minimum values of index *j*, respectively.

Weight determination–entropy weight:

The proportion of index *j* of district/county *i* is calculated as

4 $$ P_{ij} = \frac{{Y_{ij} }}{{\sum\nolimits_{i = 1}^{m} {Y_{ij} } }} $$

where *m* is the number of samples. In this research, the samples include the data of 90 districts and counties in Anhui province: thus, m = 90.

The entropy value of index *j* is solved as

5 $$ E_{j} = - 1/\ln (m)\sum\limits_{i = 1}^{m} {P_{ij} \ln (P_{ij} )} ,\;0 \le E_{j} \le 1 $$

The weight of index j is calculated as

6 $$ W_{j} = (1 - E_{j} )/\sum\limits_{j = 1}^{m} {(1 - E_{j} )} $$

Comprehensive index–multi-objective linear weighting function method:

The index score at the criterion layer s of district/county i is calculated as

7 $$ Z_{is} = \sum\limits_{i = 1}^{q} {W_{{\text{j}}} Y_{ij} } $$

where $Z_{is}$ is the index score at the criterion layer *s* of district/county *i*, and *q* is the total number of indexes at this index layer.

The total score of agricultural high-quality development level in district/county *i* is

8 $$ ESA_{i} = \sum\limits_{s = 1}^{3} {Z_{is} } $$

As per Formulas (3)–(8), the comprehensive index $ESA_{i}$ of agricultural high-quality development in 90 districts and counties of Anhui Province are acquired. A greater $ESA_{i}$ value of efficient and sustainable agriculture indicates a better efficient and sustainable agriculture level.

1. Explanatory variable. In this research, the explanatory variable $policy_{it}$ aims to measure the influence of university agricultural technology extension on efficient and sustainable agriculture.
2. Moderator variable. As previously stated, modern agricultural industrial parks are used as a moderating variable due to their status as new platforms built upon resource endowment and large-scale farming in the area. As a result, they attract modern production factors and entrepreneurs, enabling broader dissemination of agricultural policies and services. Moreover, such parks enhance the role of agricultural technology promotion in colleges and universities, contributing to more efficient and sustainable agriculture. Modern agricultural industrial parks serve as the moderator variable, which is measured based on whether a provincial-level modern agricultural industrial park is constructed.
3. Control variables. Several control variables are also selected in this research to control for the influence of other factors on efficient and sustainable agriculture. Referring to the existing relevant literature, the control variables are the logarithm of per capita disposable income of rural residents (lnincome), government financial support for agriculture (fis), crop disaster level (dis), agricultural mechanization (power), urbanization rate (urban), employment structure of rural population (emp, share of agriculture, forestry, animal husbandry and fishery in rural employment) and industrialization (indu). The disposable income per capita of the rural population reflects the ability of agricultural development to raise the income level of the rural population and influences efficient and sustainable agriculture. This index is measured by the logarithm of the per capita disposable income of rural residents. Government financial support for agricultural development is an important driver for promoting efficient and sustainable agriculture, measured by the share of total local financial expenditure on agriculture, forestry and water affairs. Uncontrollable natural phenomena and climatic conditions will have a negative impact on agricultural production. In this study, the proportion of the affected area of crops in the sown area of crops is used to indicate the level of crop disaster. Agricultural mechanization is the power source of modern agricultural development and the necessary guarantee for realizing efficient and sustainable agriculture^19^. In this study, the total power of agricultural machinery per unit of cultivated area is adopted to measure agricultural mechanization. To some extent, the urbanization rate reflects the level of economic development of a region^20^; therefore, it may affect the development of agriculture, which is measured by the urbanization rate of the population. The change in the employment structure of the rural labor force can inject new vitality into the development of regional agriculture and promote efficient and sustainable agriculture, which is reflected in the share of agriculture, forestry, animal husbandry and fishing in the rural labor force. The level of industrialization can also affect efficient and sustainable agriculture^21^, which is measured by the ratio of the output value of secondary industry to regional GDP. The change in the social and institutional environment of a county in a given year may also affect efficient and sustainable agriculture. To control for the above factors, the dummy variables for year and region are selected as control variables. Table 2 shows the associated variables and the descriptive statistical results.

**Table 2:** Variable description and descriptive statistics.

Index name	Meaning	Description	Mean	Std. Dev.	Min	Max
ESA	Comprehensive index of efficient and sustainable agriculture	Calculated by entropy method	0.355	0.125	0.058	0.803
lnincome	Per capita disposable income of rural residents	Logarithm of per capita disposable income of rural residents	9.187	0.462	7.539	10.270
fis	Government financial support for agriculture	Expenditure on agriculture, forestry, and water affairs/total financial expenditure	0.136	0.063	0.000	0.804
dis	Crop disaster level	Affected area of crops/sown area of crops	0.182	0.168	0.000	0.990
power	Agricultural mechanization	Total power of agricultural machinery/cultivated area (kW/hm^2^)	12.200	5.226	0.545	39.940
urban	Urbanization rate	urban population/total population	0.321	0.248	0.064	0.995
emp	Employment structure of rural population	Number of employees in agriculture, forestry, and animal husbandry and fishery of rural population/number of employees in rural areas	0.450	0.146	0.030	0.971
indu	Industrialization	Output value of secondary industry/GDP	0.447	0.137	0.033	0.969

## Results and discussion

### Results

#### Benchmark regression

Table 3 shows the estimated results of the influence of university agricultural technology extension on agricultural efficient and sustainable agriculture. Column (1) is the estimation result with only the fixed effect of areas and years controlled, and the coefficient at this point is 0.033, which is significant at the 1% level. Column (2) is the estimation result after adding other control variables based on column (1). In this case, the coefficient is 0.033, which is significant at the 1% level. This estimation result shows that agricultural technology extension in universities has increased the level of efficient and sustainable agriculture by 3.3%. That is, an increase of one unit in agricultural extension by the university results in a 0.033 rise in the level of both efficient and sustainable agriculture. Agricultural technology extension in universities is based on regional resources and characteristic industries. It provides various kinds of technical guidance and resource support to adjust the agricultural industrial structure, improve the efficiency of agricultural production, and promote the green and low-carbon development of agriculture. Ultimately, it achieves the goal of promoting efficient and sustainable agriculture.

**Table 3:** Impact of university agricultural technology extension on efficient and sustainable agriculture.

	(1)	(2)
Policy	0.033*** (0.009)	0.033*** (0.012)
lnincome	–	− 0.000 (0.018)
fis	–	− 0.036 (0.074)
dis	–	− 0.018* (0.010)
power	–	− 0.001 (0.001)
urban	–	− 0.012 (0.057)
emp	–	− 0.230*** (0.038)
indu	–	0.014 (0.050)
Constant	0.361*** (0.006)	0.535*** (0.146)
Year dummy	Yes	Yes
Region dummy	Yes	Yes
Observations	878	878
R^2^	0.051	0.232

***, **, and * denote significance at 1%, 5%, and 10%, respectively. Standard errors are in parentheses.

In addition, the level of crop disaster has a significant negative impact on efficient and sustainable agriculture, significant at the 10% level with a coefficient of − 0.018, and natural disasters can have a negative impact on crop planting and thus on efficient and sustainable agriculture. The employment structure of the rural population has a significant negative impact on efficient and sustainable agriculture, significant at the 1% level with a coefficient of − 0.23. suggesting that reducing the share of rural employment in agriculture, forestry, animal husbandry and fishing is conducive to promoting efficient and sustainable agriculture. Changing the initial mono-employment structure in rural areas can bring fresh blood into agriculture and promote efficient and sustainable agriculture. The effects of per capita disposable income of rural residents, government financial support for agricultural inputs, the degree of agricultural mechanisation, the urbanisation rate and the degree of industrialisation are not significant, indicating that the effects of per capita disposable income of rural residents, government financial support for agricultural inputs, the degree of agricultural mechanisation, the urbanisation rate and the degree of industrialisation are negligible after controlling for university support for agricultural technology, and also illustrating the important effects of university support for agricultural technology on the development of efficient and sustainable agriculture.

#### Parallel trend test

As shown in Table 4, when j = − 5, − 4, − 3, …, − 1, the coefficient of $policy_{it}^{ - j}$ is insignificant, indicating no significant difference in the changing trend of efficient and sustainable agriculture level between the treatment and control groups before the pilot project of agricultural technology extension in universities. Hence, the possibility of the parallel trend hypothesis cannot be rejected. In the years after the pilot agricultural technology extension in universities, the influence coefficient of $policy_{it}^{ + j}$ on efficient and sustainable agriculture is positive and significant, except for the third year. This result indicates that the agricultural technology extension in universities has a long-term promoting effect on efficient and sustainable agriculture. Moreover, the change in the coefficient indicates that its promoting effect generally shows an expanding trend. The reason may be that the effect of agricultural technology extension in universities gives farmers confidence in this policy and increases their willingness to adopt technology extension. Moreover, farmers’ ability to adopt technology is improved by early guidance and training, and the facilitating effect of agricultural technology extension in universities on efficient and sustainable agriculture is expanded accordingly.

**Table 4:** Dynamic influence of university agricultural technology extension on efficient and sustainable agriculture.

	Coefficient	Standard errors
policy^−5^	− 0.005	(0.005)
policy^−4^	− 0.007	(0.005)
policy^−3^	− 0.009	(0.010)
policy^−2^	0.001	(0.015)
policy^−1^	0.019	(0.014)
policy^+1^	0.019*	(0.010)
policy^+2^	0.025**	(0.010)
policy^+3^	0.027	(0.017)
policy^+4^	0.043***	(0.013)
policy^+5^	0.074***	(0.027)
policy^+6^	0.052***	(0.011)
lnincome	− 0.000	(0.018)
fis	− 0.035	(0.075)
dis	− 0.019*	(0.010)
power	− 0.001	(0.001)
urban	− 0.015	(0.057)
indu	0.004	(0.051)
emp	− 0.233***	(0.023)
Constant	0.540***	(0.147)
Year dummy	Yes	
Region dummy	Yes	
Observations	878	
R^2^	0.236	

***, **, and * denote significance at 1%, 5%, and 10%, respectively.

#### Robustness test

In this research, two placebo tests are used as robustness tests: the propensity score matching-difference-in-differences (PSM-DID) and the placebo test that changes the year of policy implementation and switches the treatment and control groups.

1. PSM–DID. In the benchmark regression, the DID method is used to estimate the effect of agricultural technology extension in universities on efficient and sustainable agriculture. However, this policy pilot is not an actual natural experiment; that is, there may be some differences in observable variables between pilot and non-pilot areas, leading to biased estimation results. To solve this problem, the DID method based on PSM is adopted to re-estimate the influence of university agricultural technology extension on efficient and sustainable agriculture. First, the logit model and 1:1 nearest neighbor matching method in 0.05 caliper are used in matching the propensity scores between pilot and non-pilot areas to eliminate the differences in observable covariates between them as much as possible. Then, the common support areas with propensity scores are selected for DID regression. The estimated results are shown in Column (1) of Table 5. Column (1) shows that the coefficient of $policy_{it}$ is 0.038, which is significant at the 5% level, indicating that agricultural technology extension in universities positively affects efficient and sustainable agriculture.
2. Placebo test with transformation of the treatment group. If university agricultural technology extension has a positive impact on efficient and sustainable agriculture, then the level of efficient and sustainable agriculture in the pilot areas will not be positively affected by university agricultural technology extension. Otherwise, it is doubtful whether university agricultural technology extension has a positive impact on efficient and sustainable agriculture. Therefore, a placebo test is conducted with a transformation of the treatment group: The area selected for pilot university agricultural technology extension is assumed to be a non-pilot area, i.e. a non-pilot area. Agricultural technology extension in universities does not take place in the non-pilot area, so in theory its level of efficient and sustainable agriculture should not be affected by a false attitude. To verify the above analysis, the samples of non-pilot areas are classified according to regional codes, and the areas with even regional codes are tested. Similarly, the DID method is used to estimate the effect of university agricultural technology extension on efficient and sustainable agriculture under this false setting. The estimated results are shown in Column (2) of Table 5. Column (2) shows that the coefficient of $policy_{it}$ is insignificant, proving, from a side view, that the extension of agricultural technology in universities positively affects efficient and sustainable agriculture.
3. Placebo test in dummy pilot year. The extension of efficient and sustainable agriculture level brought by agricultural technology extension in universities may be caused by other unobservable random factors unrelated to this pilot policy, leading to biased estimation results in this research. To eliminate the influence of unobservable random factors on the estimation results, the dummy pilot year placebo test is implemented: Assuming that the pilot year of agricultural technology extension in universities is one year before the actual implementation year, the DID method is also used to estimate the influence of agricultural technology extension in universities in this dummy year on efficient and sustainable agriculture. If it has no significant impact on efficient and sustainable agriculture at that time, then no unobservable random factors have interfered with the estimation results of this research, i.e. the estimation results are credible. On the contrary, it indicates that some unobservable random factors may affect efficient and sustainable agriculture, leading to the fact that the estimation results are not credible. Assuming that agricultural technology is promoted in universities in 2010, the estimated results are shown in column (3) of Table 5. Column (3) shows that the coefficient is insignificant, indicating that no unobservable random factors have interfered with the estimation results of this research. This means that agricultural technology extension in universities has a positive impact on efficient and sustainable agriculture.

**Table 5:** Robustness test.

	(1)	(2)	(3)
	PSM–DID	Placebo test with transformation of the treatment group	Placebo test in dummy pilot year
Policy	0.038*** (0.013)	0.008 (0.010)	0.017 (0.010)
lnincome	− 0.005 (0.018)	0.004 (0.018)	0.004 (0.018)
fis	0.072 (0.062)	− 0.045 (0.076)	− 0.047 (0.074)
dis	− 0.016* (0.010)	− 0.017* (0.010)	− 0.018* (0.010)
power	0.002* (0.001)	− 0.001 (0.001)	− 0.001 (0.001)
urban	− 0.033 (0.046)	− 0.025 (0.056)	− 0.020 (0.057)
emp	− 0.255*** (0.043)	− 0.228*** (0.038)	0.022 (0.050)
indu	− 0.027 (0.051)	0.028 (0.048)	− 0.229*** (0.038)
Constant	0.567*** (0.153)	0.496*** (0.152)	0.503*** (0.149)
Year dummy	Yes	Yes	Yes
Region dummy	Yes	Yes	Yes
Observations	774	878	878
R^2^	0.260	0.219	0.219

***, **, and * denote significance at 1%, 5%, and 10%, respectively. Standard errors are in parentheses.

#### Mechanism analysis and the modulating effect of modem agricultural industrial parks

To analyze the mechanism of action of agricultural technology extension in universities in promoting efficient and sustainable agriculture, the influence of agricultural technology extension in universities on coordinated, efficient and green agricultural development is estimated. The estimated results in columns (1)–(3) of Table 6 show that agricultural technology extension in universities has a significantly positive effect on coordinated and green agricultural development. However, the regression coefficient for efficient development is insignificant, indicating that agricultural technology extension in universities promotes efficient and sustainable agriculture by promoting the coordinated and green development of agriculture. On the one hand, agricultural technology extension in universities facilitates the coordinated development of agriculture by combining the planting of local characteristic agricultural products. Thus, it contributes to the creation of local characteristic agricultural product brands through special technology research and development and promotes the planned adjustment of the internal structure of agriculture under the premise of ensuring food security. On the other hand, the development of agricultural technology in universities also promotes the development of agricultural auxiliary industries, such as agricultural product processing, storage and logistics, and increases the output value of agricultural auxiliary activities. The facilitating effect of university agricultural technology extension on the green development of agriculture can reduce the consumption of resources and the destruction of nature by agricultural production through the extension of green agricultural technologies, such as the reduction of fertilizers and the green control of diseases and pests. Conversely, the cultivation of crops with better traits can reduce the subsequent consumption of resources to ensure crop growth. The extension of agricultural technology in universities has not had a significant impact on the efficient development of agriculture. This may be because agricultural production activities have a long cycle and are strongly influenced by the natural environment. The improvement in agricultural production efficiency brought about by new technologies and methods is not obvious in a short period of time.

**Table 6:** Mechanism analysis and the modulating effect of modem agricultural industrial parks.

	(1)	(2)	(3)	(4)
	Coordinated development	Efficient development	Green development	ESA
Policy	0.021*** (0.006)	0.006 (0.009)	0.006* (0.003)	0.030** (0.013)
Policy*AP	–	–	–	0.025** (0.010)
lnincome	− 0.019* (0.009)	− 0.002 (0.016)	0.020*** (0.005)	0.000 (0.018)
fis	0.035 (0.034)	− 0.082 (0.055)	0.012 (0.018)	− 0.034 (0.074)
dis	− 0.007 (0.005)	− 0.008 (0.007)	− 0.004 (0.004)	− 0.018* (0.010)
power	0.000 (0.000)	− 0.000 (0.001)	− 0.001*** (0.000)	− 0.001 (0.001)
urban	− 0.040** (0.019)	− 0.035 (0.039)	0.064*** (0.011)	− 0.015 (0.057)
emp	0.003 (0.013)	− 0.201*** (0.038)	− 0.032*** (0.007)	− 0.231*** (0.038)
indu	− 0.039* (0.023)	− 0.002 (0.041)	0.055*** (0.013)	0.012 (0.050)
Constant	0.268*** (0.080)	0.310** (0.132)	− 0.042 (0.040)	0.534*** (0.146)
Year dummy	Yes	Yes	Yes	Yes
Region dummy	Yes	Yes	Yes	Yes
Observations	878	878	878	878
R^2^	0.256	0.331	0.546	0.234

***, **, and * denote significance at 1%, 5%, and 10%, respectively. Standard errors are in parentheses.

Modern agricultural industrial parks are an important carrier for gathering essential resources, innovating institutional mechanisms and promoting the development of modern agriculture. Since the establishment and accreditation of the National Modern Agricultural Industrial Park in 2017, a great deal of exploration and practice has been carried out in the establishment of modern agricultural industrial parks across the country. Moreover, remarkable achievements have been made in the construction of the whole agricultural industrial chain, the establishment of the mechanism of linking agriculture and industry, the extension of the transformation of agricultural production mode, and the acceleration of the innovation of agricultural science and technology, which has effectively promoted efficient and sustainable agriculture everywhere^22^. This study examines the role of modern agricultural industrial parks in the extension of agricultural technology in universities and the extension of efficient and sustainable agriculture. In column (4) of Table 6, the interactive term between university agricultural technology extension and modern agricultural industrial parks is added based on the benchmark regression. The regression result is significantly positive at the 5% level, indicating that modern agro-industrial parks exert a positive moderating effect that can strengthen the facilitating effect of university agro-technology extension on efficient and sustainable agriculture. Through the aggregation effect of modern agro-industrial parks, university agro-technology extension can promote a wider range of activities, such as technology extension and farmer training. In this way, the scope and influence of university agricultural technology extension will be expanded, the beneficiary groups of university agricultural technology extension will be enlarged, and the role of university agricultural technology extension in promoting efficient and sustainable agriculture will be enhanced as a whole.

#### Heterogeneity analysis

To explore the heterogeneity of the influence of university agricultural technology extension in different regions on efficient and sustainable agriculture, regression analysis is conducted on the southern, central and northern regions of Anhui Province using the DID method. The estimated results in columns (1)–(3) of Table 7 show that university agricultural technology extension has a significantly positive effect on efficient and sustainable agriculture in the southern and central regions but has no significant effect in the northern regions. The reason for this difference may be that, compared with the northern region, the southern and central regions have a better economic environment and better agricultural infrastructure; thus, these regions can better integrate the resources of all sectors of society and provide the basic conditions for efficient and sustainable agriculture in the process of agricultural technology extension in universities. However, the traditional agricultural development model in the northern region is deeply rooted, with a large proportion of agricultural carbon emissions but low agricultural ecological efficiency, unreasonable inputs of various factors and unbalanced development^23^. The effect of agricultural technology extension in universities will take a long time.

**Table 7:** Heterogeneity analysis.

	Different regions			Different development levels		
	(1)	(2)	(3)	(4)	(5)	(6)
	Southern region	Central region	Northern region	Low	Medium	High
Policy	0.067*** (0.021)	0.050*** (0.014)	0.004 (0.016)	0.023 (0.016)	0.012 (0.022)	0.053*** (0.016)
lnincome	− 0.070 (0.063)	0.018 (0.025)	0.026 (0.031)	0.014 (0.020)	− 0.013 (0.018)	− 0.078 (0.056)
fis	− 0.106 (0.147)	0.083 (0.136)	0.034 (0.083)	− 0.043 (0.060)	− 0.100 (0.076)	− 0.121 (0.152)
dis	− 0.018 (0.019)	0.027 (0.018)	− 0.033** (0.012)	− 0.016* (0.008)	− 0.005 (0.008)	0.014 (0.015)
power	− 0.001 (0.002)	− 0.002 (0.003)	− 0.001 (0.002)	− 0.003* (0.001)	0.001 (0.001)	0.001 (0.001)
urban	− 0.167 (0.123)	0.078 (0.057)	0.008 (0.089)	0.017 (0.067)	− 0.023 (0.053)	0.064 (0.069)
emp	− 0.344*** (0.093)	− 0.177*** (0.063)	− 0.188*** (0.047)	− 0.161*** (0.029)	− 0.070* (0.036)	− 0.158** (0.071)
indu	0.031 (0.107)	0.055 (0.097)	0.034 (0.055)	0.078** (0.038)	− 0.080 (0.054)	0.172** (0.066)
Constant	1.283** (0.505)	0.342* (0.192)	0.198 (0.270)	0.282 (0.169)	0.571*** (0.150)	1.175** (0.481)
Year dummy	Yes	Yes	Yes	Yes	Yes	Yes
Region dummy	Yes	Yes	Yes	Yes	Yes	Yes
Observations	299	286	292	332	273	273
R^2^	0.374	0.369	0.316	0.355	0.171	0.273

***, **, and * denote significance at 1%, 5%, and 10%, respectively. Standard errors are in parentheses.

To explore the heterogeneity of the influence of agricultural technology extension on efficient and sustainable agriculture in regional universities with different levels of efficient and sustainable agriculture, the DID method is also used to perform regression analysis on three groups of regions with low, medium, and high levels of efficient and sustainable agriculture. The estimated results in Columns (4)–(6) of Table 7 show that the extension of agricultural technology in universities has a significantly positive influence on areas with highly efficient and sustainable agriculture level, but not on areas with low or medium level. A possible reason is that in the areas with highly efficient and sustainable agriculture levels, university agricultural technologies can be popularized and applied to actual production more quickly. In comparison with the farmers in the areas with low efficient and sustainable agriculture level, the comprehensive quality of farmers in such areas is generally higher, and they have higher digestion and absorption ability for new technologies and methods. After training, guidance, and demonstration, they can use the achievements from agricultural technology extension in a short time, thereby improving the agricultural quality development level. For areas with low levels of efficient and sustainable agriculture due to their weak development strength, weak acceptance ability, and low application efficiency for the same extension technology, applying new methods and technologies to solve problems in practice in a short time is difficult. Thus, the effect of agricultural technology extension in universities is not significant for such areas.

### Discussion

#### Baseline regression results

The results of the baseline regression indicate that university agricultural technology extension has increased the level of efficient and sustainable agriculture by 3.3%. Based on regional resources and special industries, university agricultural technology extension provides various kinds of technical guidance and resource support to regulate the structure of agricultural industry, improve agricultural production efficiency, and promote green and low-carbon agricultural development, ultimately achieving the goal of promoting efficient and sustainable agriculture. Existing research indicates that the implementation of new technologies significantly contributes to the high-quality and sustainable development of industries^12^. This study on agricultural technology extension in universities establishes that it enhances both the efficiency and sustainability of agricultural production. Correspondingly, by providing guidance and demonstration, technology extension programmes implemented by universities can assist farmers in employing novel technologies to achieve efficient and sustainable production. In addition, the level of crop damage has a significant negative impact on efficient and sustainable agriculture, with natural disasters having a negative impact on crop production, which in turn has a negative impact on efficient and sustainable agriculture. The employment structure of the rural population has a significant negative impact on efficient and sustainable agriculture, suggesting that reducing the proportion of rural workers employed in agriculture, forestry, animal husbandry and fishing is conducive to efficient and sustainable agriculture, and that changing the initially homogeneous employment structure in rural areas can bring new blood into agriculture and promote efficient and sustainable agriculture.

#### Analysis of the mechanism and the moderating effect of modern agro-industrial parks

The results in Table 6 show that university agricultural technology extension promotes efficient and sustainable agriculture by fostering coordinated and green agricultural development. The role of university agricultural extension in promoting coordinated agricultural development is, on the one hand, due to the fact that university agricultural extension incorporates the local special agricultural farming industry, helps to build local brands of special agricultural products through special technology research and development, and promotes internal agricultural restructuring in a planned manner while ensuring food security; on the other hand, university agricultural extension also promotes the development of agricultural auxiliary industries, such as agricultural products On the other hand, university agricultural extension also promotes the development of ancillary agricultural industries, such as the processing industry, storage, logistics, etc., and increases the output value of ancillary agricultural activities. On the one hand, it reduces the consumption of resources and damage to nature through the extension of green agricultural technologies such as fertilizer and pest control, and on the other hand, it reduces the subsequent consumption of resources to ensure the growth of crops by producing crops with better traits. The lack of significant promotion of efficient agricultural development by the University's agricultural technology diffusion may be due to the long cycle of agricultural production activities and the fact that they are highly influenced by the natural environment, and the increase in agricultural production efficiency brought about by new technologies and methods is not obvious in a short period of time. This mechanism of action has not been discussed in existing studies, which have discussed how knowledge-based agriculture can improve sustainable productivity and product quality for sustainable agriculture by monitoring crop quality and yield assessment through modern computer technology^9^, while some studies have used nanotechnology as an example of a mechanism of action to explore the impact of agricultural technology on sustainable agriculture by illustrating that the development of nano-pesticides using nanocarriers can increase the biological activity of synthetic or natural (plant) pesticides while reducing their adverse effects on the environment as a mechanism of action to explore the impact of agricultural technology on sustainable agriculture^11^. However, this study is different from previous studies in that it illustrates the specific mechanism of action by exploring the impact of university agricultural technology extension on specific dimensions of efficient and sustainable agriculture, and it is found that the impact of university agricultural technology extension on efficient and sustainable agriculture is, on the one hand, to improve the efficiency of agricultural production by adjusting the internal structure of agriculture, and on the other hand, to maintain the sustainability of development by reducing consumption and improving crops to ultimately realise efficient and sustainable agriculture, and this mechanism discussion is somewhat innovative.

Modern agricultural industrial parks are an important carrier for gathering factor resources, innovating institutional mechanisms and promoting the development of modern agriculture. Since the establishment and recognition of national modern agricultural industrial parks was launched in 2017, a great deal of exploration and practice has been carried out in the establishment of modern agricultural industrial parks across the country, and significant results have been achieved in creating whole agricultural industrial chains, building mechanisms to link and guide farmers, promoting the transformation of agricultural production methods, and accelerating innovation in agricultural science and technology, which have strongly promoted efficient and sustainable agriculture in various regions^22^. This paper examines the role of modern agricultural industrial parks in the process of promoting efficient and sustainable agriculture through university agricultural technology diffusion. Column (4) of Table 6 adds the interaction term between university agricultural extension and modern agricultural industrial parks to the baseline regression, and the regression results are significantly positive at the 5% level, indicating that there is a positive moderating effect of modern agricultural industrial parks, which can strengthen the role of university agricultural extension in promoting efficient and sustainable agriculture. With the aggregation effect of modern agricultural industrial parks, university agricultural extension can conduct a wider range of technical extension and farmer training activities, thus expanding the coverage and influence of university agricultural extension, and increasing the number of beneficiaries of university agricultural extension, which ultimately enhances the promotion of university agricultural extension for efficient and sustainable agriculture in general. This is in contrast to previous studies which have focused on how agricultural technology affects specific agricultural production and which parts of production it affects, while this study explores how, in promoting efficient and sustainable agriculture through university agricultural technology extension, modern agricultural industrial parks can enhance this promotion through the platform's convergence leadership.

#### Analysis of heterogeneity test results

The differences between the three regions of southern, central and northern Anhui may be due to the fact that the economic environment and agricultural infrastructure in southern and central Anhui are better than those in northern Anhui, and they can better integrate the resources of the community into the agricultural development process and provide the basic conditions for efficient and sustainable agriculture. In contrast, the traditional agricultural development model in northern Anhui is deeply rooted, with a high proportion of agricultural carbon emissions but low agricultural eco-efficiency, unreasonable input of various factors, and uncoordinated and unbalanced development, and it will take a longer period of time before the role of university agricultural technology extension can be brought into play.

The difference in the results of different levels of development of efficient and sustainable agriculture may be due to the fact that areas with a high level of efficient and sustainable agriculture are able to apply university agricultural technologies more quickly in production, as the overall quality of farmers in these areas is generally higher compared to farmers in areas with a lower level of development, and they have a higher capacity to digest and absorb new technologies and methods, and are able to maturely apply the results from agricultural technology extension in a shorter period of time after training, guidance and demonstration. They are able to use the results from agricultural extension in a mature manner within a relatively short period of time after training, guidance and demonstrations, thus improving the level of efficient and sustainable agriculture. For areas with a low level of efficient and sustainable agriculture, the effectiveness of university agricultural extension is not significant due to the weakness of their own development, and their weak ability to accept and apply the same extension techniques, making it difficult to apply new methods and techniques in practice in a short period of time. Existing research is less for sub-region and development level to discuss the role of university agricultural technology extension on efficient and sustainable agriculture, which is better for the university agricultural technology promotion to play an important role in the impact of agricultural development, this study through the differences in the discussion, we can improve the university agricultural technology extension to adapt to the specific conditions of different regions to realise the effect of its promotion of efficient and sustainable agricultural development.

## Conclusions

Based on the quasi-natural experiment of university agricultural technology extension conducted by Anhui Agricultural University after 2012, and using the county-level panel data from 2008 to 2020, this research empirically analyses the influence of university agricultural technology extension on efficient and sustainable agriculture. The conclusions are as follows. First, the extension of agricultural technology in universities has a positive effect on efficient and sustainable agriculture, which increases the comprehensive index of efficient and sustainable agriculture level by 3.3%. Universities utilise their own resources, including human capital and technological research and development capabilities, to advance scientific and technological achievements in agriculture. They provide guidance to local agricultural production activities to enable practical application of agricultural technology, leading to enhanced productivity and sustainable, environmentally conscious production methods, thus achieving efficiency and sustainability in agriculture. Second, the extension of agricultural technology in universities can promote efficient and sustainable agriculture by influencing the coordinated and green development dimensions in efficient and sustainable agriculture. On one hand, university agricultural technology extension brings together local specialty agricultural plantations and promotes coordinated development by adjusting the internal structural adjustment of agriculture through specialty technology guidance. On the other hand, it employs green agricultural technology and improved varieties to foster environmentally conscious development. Third, agricultural industrial parks have a moderating effect on the extension of agricultural technology in universities and efficient and sustainable agriculture. The presentation and influential impact of modern agricultural industrial parks have the potential to broaden the scope of agricultural technology extension in universities. Fourth, the extension of agricultural technology in universities has a significant positive effect on the development of high-quality agriculture in the southern and central areas of Anhui Province, but not in the northern areas. Fifth, the pilot project of agricultural technology extension in universities has a significant positive effect on areas with a high level of efficient and sustainable agriculture, but it has no significant effect on areas with a low and medium level.

The policy implications of this research are mainly fourfold. First, the agricultural technology extension system in universities should be pursued and improved. Agricultural technology extension in universities can improve the quality of farmers through experiments, demonstrations, farmer training and technical support. In addition, agricultural science and technology should be popularized to inject vitality into agricultural development, thereby promoting efficient and sustainable agriculture. Second, the role of university agricultural technology extension in promoting efficient agricultural development is not significant. In the future, university agricultural technology extension services should pay more attention to technical research on improving agricultural production efficiency, reducing unnecessary human and material consumption in the agricultural production process, and realizing the optimal allocation of input factors. Third, modern agricultural industrial parks can enhance the facilitating effect of agricultural technology extension in universities on efficient and sustainable agriculture. Once established, modern agricultural industrial parks provide a better platform for agricultural technology extension in universities. With the radiation of this modern agricultural development platform, it can spread the training and demonstration role of agricultural technology extension in universities to a greater extent and strengthen its effect in promoting efficient and sustainable agriculture. Fourth, there are obvious differences in the effects of agricultural technology extension in universities on efficient and sustainable agriculture in different regions. Before technology extension, the problems faced by local farmers in agricultural production should be fully understood, and targeted research on key problems should be conducted. Introduce new technologies into agricultural production practices. Provide focused instructions on soil amelioration and fertilisation techniques for regions with poor soil quality. Allocate additional technical extension staff in regions with rudimentary and unsustainable farming practices to offer comprehensive guidance. Better extension is achieved by adapting the type of extension technology and the scale of extension to different regions at the time of extension.

## Data Availability

All data generated or analyzed during this study are included in this published article.

## Abbreviations

CCP: The Central Committee of the Chinese Communist Party
DID: Difference-in-differences
ESA: The comprehensive index of efficient and sustainable agriculture
lnincome: Per capita disposable income of rural residents
fis: Government financial support for agriculture
dis: Crop disaster level
power: Agricultural mechanization
urban: Urbanization rate
emp: Employment structure of rural population
indu: Industrialization
PSM-DID: The propensity score matching-difference-in-differences
PSM: The propensity score matching
AP: The National Modern Agricultural Industrial Park
